# Joseph Constantine Carpue and the Bicentennial of the Birth of Modern Plastic Surgery

**DOI:** 10.1093/asj/sju157

**Published:** 2015-03-20

**Authors:** M. Felix Freshwater

**Affiliations:** Dr Freshwater is a Voluntary Professor of Surgery at the University of Miami School of Medicine, Miami, FL

## Abstract

September 2014 marked the bicentennial of the birth of modern plastic surgery. It was then that Carpue began a prospective observational study of nasal reconstruction that culminated in his 1816 monograph, which caused an explosion of interest in reconstructive surgery throughout Europe. In conducting his study, Carpue demonstrated ethical standards and the power of planning a procedure. His methods to document his results accurately would remain unsurpassed until photography was adopted at the end of the 19th century. Carpue took an apocryphal story of surgery performed in India more than twenty years earlier and transformed it into the beginning of modern plastic surgery. He succeeded in a number of unrecognized tasks that are themselves landmarks not only in plastic surgical history, but surgical history: devising the first prospective observational study, using exclusion criteria, maintaining appropriate patient confidentiality, setting a standard for preoperative disclosure and ethical approval over a century before these measures were codified, having independent documentation of his preoperative and postoperative findings, devising a method to objectively monitor and document the forehead flap, and describing the potential value of tissue expansion. He shared his experience by publishing his results and by lecturing in Europe. His contemporaries recognized him for his contributions and he was honored by election to the Royal Society. Carpue launched the modern era of plastic surgery in an ethical, logical, and objective manner. While plastic surgery has changed in the last two centuries, the principles that Carpue followed remain valid.

Most modern plastic surgeons recognize the name Gaspare Tagliacozzi even if they cannot pronounce it properly. The American Board of Plastic Surgery's (ABPS) seal contains Tagliacozzi's face and the American Association of Plastic Surgery's (AAPS) seal depicts a patient immobilized in a jacket while his arm flap is provided for nasal reconstruction from his 1597 book *De Curtorum Chirgica per Institionem*. Jerome P. Webster, who was a founding member of ABPS and longtime officer of AAPS, created both seals.^1^

Martha Gnudi and Webster wrote the definitive biography of Tagliacozzi for which they won the Welch Medal from the American Association of the History of Medicine in 1954.^2^ They concluded that Tagliacozzi's historical importance was not because he developed anything new, but because he shared knowledge.^3^ In his 1597 book, Tagliacozzi described techniques that the Branca family had developed yet kept secret for more than a century. Tagliacozzi's book, coming as it did from a professor of medicine at the University of Bologna, gave it some importance. Nevertheless, after he died in 1599, his methods were sealed as if in a sarcophagus. They were not to be unearthed for over two centuries when Joseph Constantine Carpue described them. Indeed, a letter to John Fulton, Chairman of the History of Medicine at Yale, by Webster is revealing: “[It was] Joseph Constantine Carpue, who brought plastic surgery back to Europe after more than 200 years, Tagliacozzi's work having died out.”^4^

This is the story of Carpue, a surgeon-scientist and his place in the history of plastic surgery. He conducted his own original clinical research two centuries ago, and he can be considered the father of modern plastic surgery.

## CARPUE'S EARLY LIFE AND EDUCATION

Joseph Constantine Carpue was born in May 1764 in Brook Green, which still is an affluent neighborhood in London, England. His grandfather, Charles, had made the family fortune as a shoe manufacturer and his uncle, William Lewis, was a leading publisher in London.^5^ Carpue was “a late bloomer”; for more than a decade after leaving the Jesuit college of Douai he vacillated about his future. Perhaps this was a manifestation of his innate curiosity, for he explored a host of careers. As a Catholic, Carpue first thought of becoming a priest; next he toyed with the idea of joining his uncle's publishing business. The law appealed to him briefly, as the 1791 Roman Catholic Relief Act allowed Catholics to join the legal profession. He was next “smitten with admiration for Shakespeare” and considered a career on the stage.^6^ Finally, on August 5, 1796, Carpue registered at St. George's Hospital Medical School for a one-year term. Carpue was an unusual student for two reasons, first, he enrolled when he was 32 years old, and second, he was a college graduate, which was the exception to the rule for proto-surgeons of the 18th century. He studied under surgeon Everard Home, John Hunter's brother-in-law and successor at St. George's.

## CARPUE'S EMPLOYMENT AND EARLY CAREER

In the late 18th century, surgery was still rife with nepotism and, as such, Carpue's professional prospects were limited. His abilities were known to Home, who offered him £500 a year to serve as his assistant.^7^ It is unknown if Carpue accepted Everard Home's offer, but in 1799, Carpue become a staff-surgeon at the Duke of York's Hospital, a military hospital in Chelsea.^8^ He accomplished this through the influence of Thomas Keate, whom he had known as a surgeon at St. George's, and who was surgeon general of the Army.^7^ No records remain describing the patients Carpue treated while at Chelsea before he resigned in 1807.

**Figure 1.**
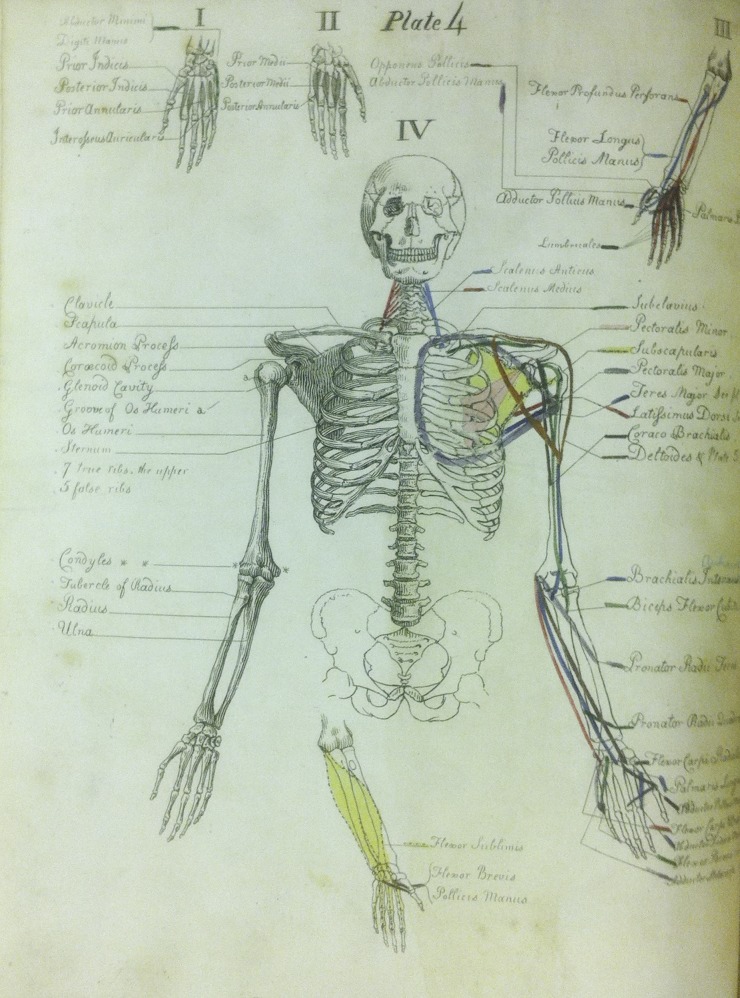
Hand-colored illustration from Carpue's “A Description of the Muscles of the Human Body.”^11^ Note how Carpue designed the colors to represent the muscles' origins and insertions. Carpue was known as “The Chalk Professor”. No other artist was credited with the drawings in contrast to Turner who was credited with the illustrations in Carpue's 1816 book. Courtesy of the National Library of Medicine.

In 1800, Carpue tutored George Norman to prepare for the Royal College of Surgeons' examination. Norman had only his prior medical education of an apprenticeship with his father, a surgeon in Bath.^9^ Norman had approached Carpue and said, “I wish I knew anatomy as well as you, Carpue.” After Norman passed his M.R.C.S. examination on June 4, 1801, he insisted that Carpue accept 20 guineas as payment.^10^ This spurred Carpue to present formal classes in anatomy and surgery. Later that year, Carpue confirmed his reputation as an anatomist and teacher by publishing “*A Description of the Muscles of the Human Body as They Appear on Dissection”* that was self-illustrated*.*^11^ (Figure 1) Drawing was part of his unique teaching style; he was thought to have been the first anatomy instructor to draw diagrams while demonstrating anatomy, which resulted in his nickname “The chalk professor.” Carpue's classes proved to be popular not only with students preparing for their fellowship examination, but also with aristocrats, members of parliament, barristers, and law students.^7^

Carpue's interest in muscles sparked his curiosity about galvanism. He performed several experiments with Galvani's nephew, Luigi Aldini. In 1803, Carpue published “*Introduction to Electricity and Galvanism, with cases shewing their effects in the cure of diseases; to which is added, a description of Mr. Cuthbertson's plate electrical machine.”* While the title would suggest that Carpue was favorably disposed to these methods, what was particularly noteworthy was Carpue's objectivity noting both failures and successes. He began his description thusly: “I shall read a number of cases, as well those in which *I have been unsuccessful*, as those wherein I have succeeded.” [italics added].^12^

## CARPUE'S PROSPECTIVE OBSERVATIONAL STUDY

**Figure 2.**
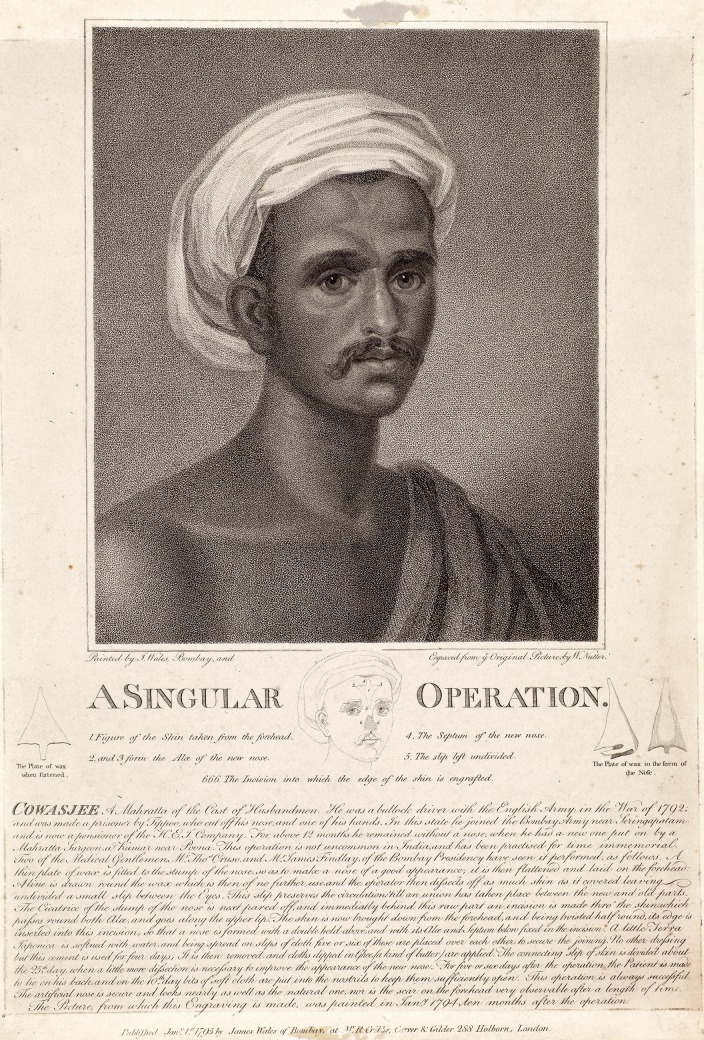
“A Singular Operation” engraving by William Nutter after a painting by James Wales, London, January 1, 1795. Courtesy of the Wellcome Library, London.

To understand the context of Carpue's prospective study, consider these facts:

1. Anesthesia, asepsis, and antibiotics did not exist in 1814.
2. John Heaviside referred an army officer who had been under his care for five years. Heaviside believed that the patient lost his nose from eight years of mercury ingestion rather than from venereal disease.^11 (pp81-2)^
3. With his Jesuit education, Carpue could read Tagliacozzi's Latin book.
4. Search indexes did not exist at the time. Hand searching the medical literature for a description of forehead flap nasal reconstruction would not have been fruitful. The primary source of information about the procedure was an engraving based upon the original painting of an Indian nasal reconstruction.^13,14^ (Figure 2).

Carpue described his prospective study on this and a second patient in meticulous detail when, in 1816 at the age of 49, he published his monograph: “*An account of two successful operations for restoring a lost nose with the integuments of the forehead in the cases of two officers of His Majesty's army, in which are included historical and physiological remarks on the nasal operation including descriptions of the Indian and Italian methods.*”^15^

## CARPUE –— IN HIS OWN WORDS

Rather than attempt to convey the meaning of what Carpue accomplished and how he did so, it is better to see what he wrote in his own words. Table 1 lists the noteworthy elements of his work.

**Table 1.** Carpue's Accomplishments

1	Prospective observational study
2	Informed consent
**3**	Ethical approval
**4**	Exclusion criteria
**5**	Operative planning
**6**	Patient confidentiality
**7**	Preoperative documentation
**8**	Tissue expansion
**9**	Postoperative monitoring
**10**	Independent postoperative documentation

### Pretreatment Disclosure

Carpue's first patient had thought that Carpue had previously done a nasal reconstruction. Carpue's description of their meeting in September 1814, mirrors modern informed consent in its forthrightness and disclosure of dangers and alternative operations:

I readily consented; but, at the same time, apprized my patient, that what he had previously heard, was founded in mistake. I had long wished for an opportunity of performing the operation; and, for the space of fifteen years, had constantly recommended it to my pupils. I added, that I considered it as by no means dangerous, and that it might be practiced in either of two methods: the one, the Italian, or, as it is commonly called, the Taliacotian, in which the part is supplied from integuments of the arm; the other, the Indian, in which it is taken from the forehead.^15(pp81-2)^

### Ethical Approval

Now we have ethics committees and the Declaration of Helsinki for guidance on experimental procedures. Article 37 of the latter states, in part: “Where proven interventions do not exist or have been ineffective, the physician, after seeking expert advice, with informed consent from the patient … , may use an unproven intervention if in the physician's judgement it offers hope of saving life, re-establishing health or alleviating suffering.”^16^

Carpue gave the best available proof of concept and obtained assent from others for his proposed procedure as follows: “[I] performed the operation in my theatre on the dead subject, before my pupils, and a number of medical friends. [They] unanimously agreed to the propriety of the operation.”^15(p84)^

### Exclusion Criteria

How do we know that Carpue believed that he was conducting a prospective study? Carpue actually did the noun trial and specified an exclusion criterion. Carpue had received assurances from two independent surgeons, Heaviside and John Pearson, that the nasal loss was from “the injudicious use of mercury” rather than from syphilis and he published their certificates in his book's appendix.^15(pp99-100)^ Carpue was wary that his patient's healing abilities had been impaired, and he needed visual proof that his patient did not have problems healing wounds: “The question to be decided was, is this a fair case for *trial* … I wished to determine the point; and therefore, under pretext of preparing for the operation, I made incisions near the remains of the alae. The wounds healed.” [italics added]^15(p83)^

### Patient Confidentiality

Despite these assurances and the patient's healing ability, Carpue may have suspected that syphilis was the root of the nasal loss, as he never disclosed his first patient's name. In contrast, Carpue named his second patient who was a war hero and who had sustained his nasal loss in battle. This patient had been referred to Carpue by George IV when he was the Prince of Wales. George IV paid all of this patient's medical expenses, and Carpue dedicated his book to the Prince.^15(pp2nd title p, 96-7)^

### Operative Planning

How did Carpue plan a procedure that he had never seen or done? He wrote: “I commenced a series of experiments on the dead … I operated in that manner eleven times … I received much assistance in planning and executing this new operation, and its stages.”^15(p84)^

### Pretreatment Documentation

**Figure 3.**
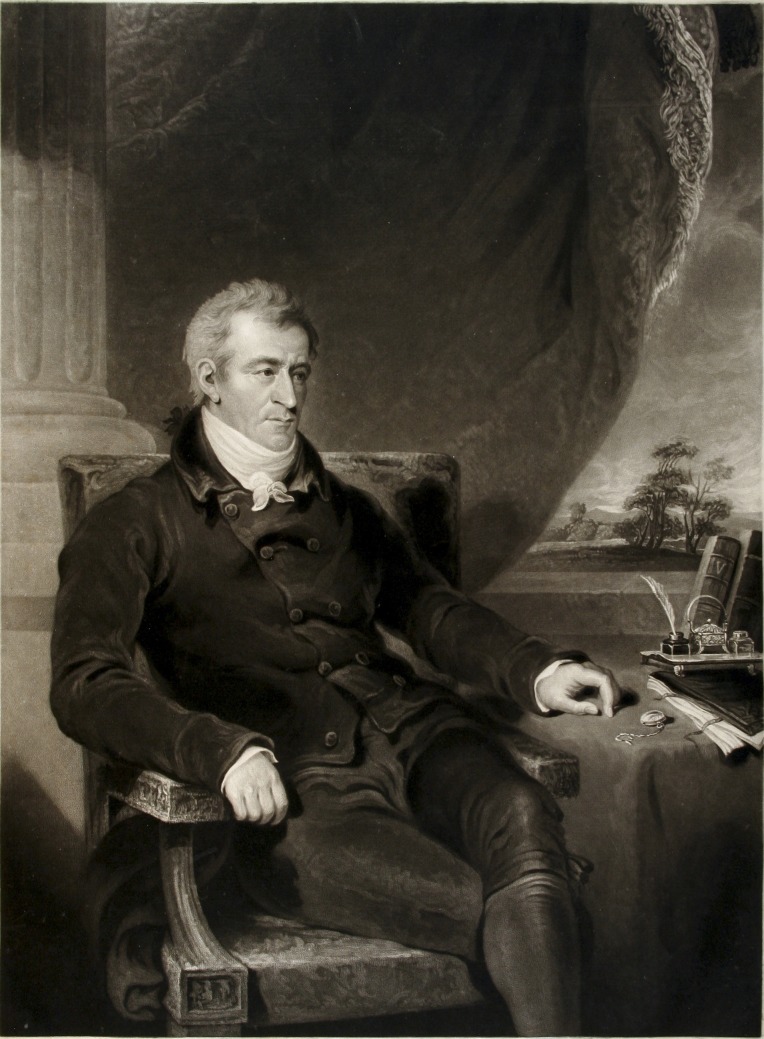
Joseph Constantine Carpue engraving by Charles Turner. Turner was the foremost engraver in England whose engravings of J.M.W. Turner's paintings opened them to popular consumption. Courtesy of the Wellcome Library, London.

**Figure 4.**
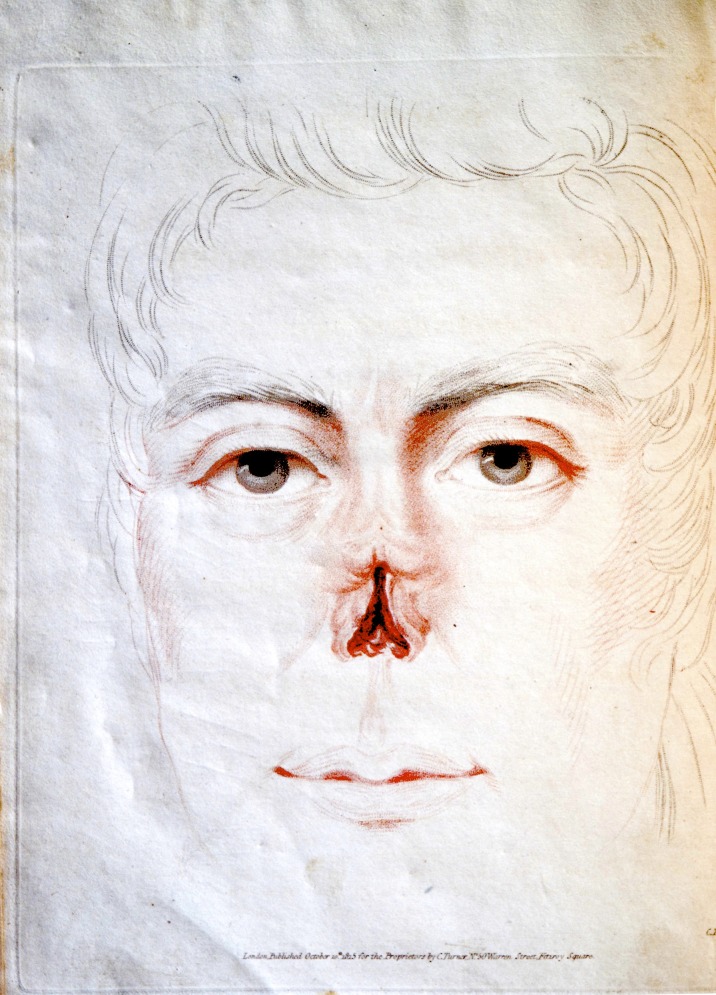
Plate 2 from Carpue 1816, engraving by Charles Turner. This hand-colored engraving shows Carpue's first patient's deformity with the distal third of the nose including the septum being missing. Courtesy of the Wellcome Library, London.

Although Carpue was a talented artist who had drawn his own illustrations for his first book, he had illustrations for this book made by Charles Turner, the mezzotint engraver best known for his engravings of J.M.W. Turner's paintings. Carpue and Turner would have more than a professional relationship, with Carpue giving Turner's son the gift of his working copy of Tagliacozzi's book.^17^ (Figures 3 and 4).

### Tissue Expansion

Carpue did the first stage on October 23, 1814, without any anesthesia; it took 15 minutes.^15(p86)^ By October 27, Carpue was concerned that his nasal reconstruction lacked sufficient projection, he considered correcting this by fabricating a device based upon the available technology, but this did not prove to be necessary: “The flatness of the nose alarmed [me] I thought of procuring the air-bladder of a fish, … to introduce into the nose, and then inflate, with the design of raising the point of the nose.”^15(p88)^

### Objective Serial Postoperative Flap Monitoring

To quantify nasal projection over the course of time in an objective manner, Carpue devised a simple, yet elegant means: “I … [placed the patient to allow] his profile to be drawn upon a wall; and, by making the comparison every two or three days, I had the pleasure to see its gradual increase demonstrated.”^15(p90)^

### Honest and Independent Postoperative Documentation

**Figure 5.**
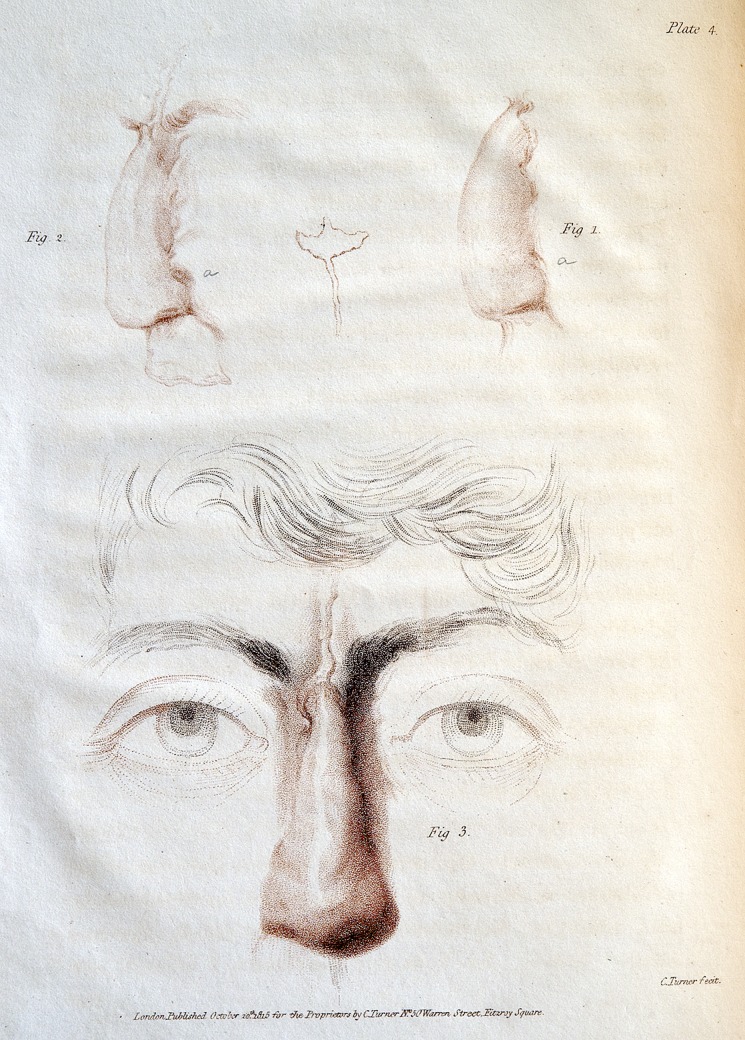
Plate 4 from Carpue 1816, engraving by Charles Turner. This hand-colored engraving shows three views of Carpue's first patient's postoperative result, lateral, oblique, and frontal. Note the fistula marked as ‘a’ in the lateral and oblique views. Courtesy of the Wellcome Library, London.

Carpue described a fistula that developed and documented its location in his figures.^15(p86)^(Figure 5) This showed a degree of accuracy that would not be exceeded until Keegan published engravings based on photographs of nasal reconstructions with forehead flaps later in the 19th century.^18^

## CARPUE'S CONTEMPORARY RECOGNITION AND RELEVANCE

**Figure 6.**
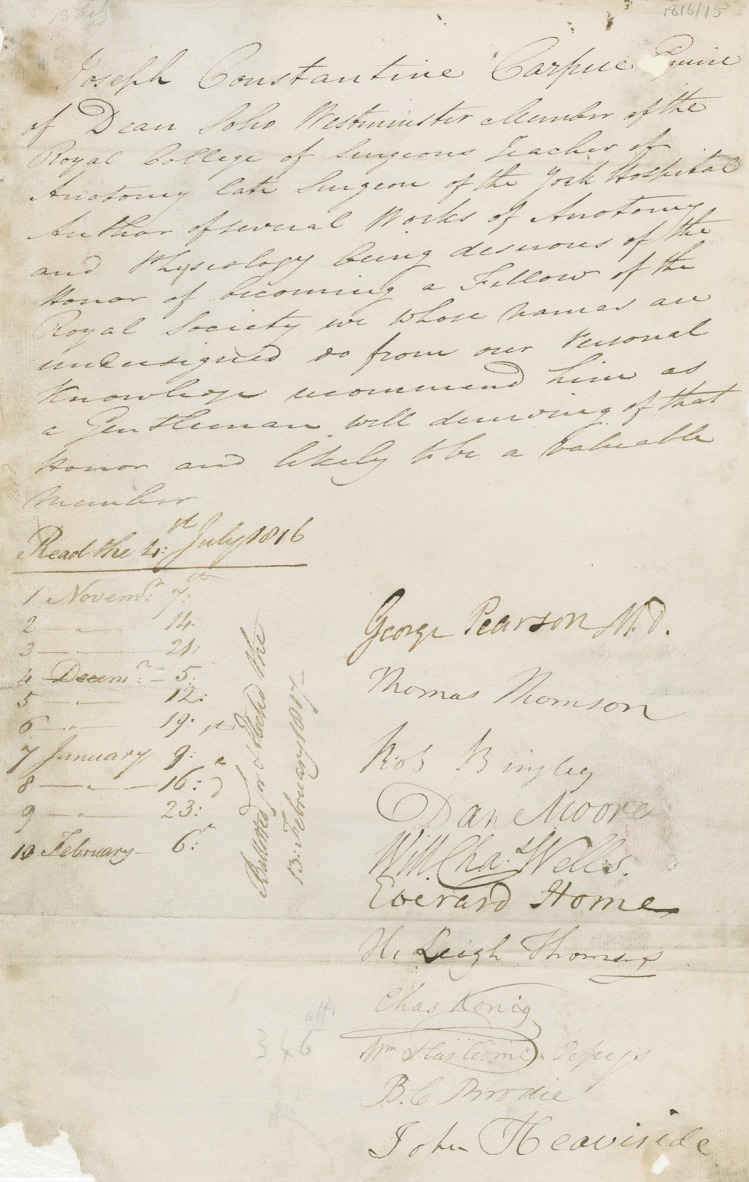
This is a copy of the certificate for Carpue's election to the Royal Society. It was signed by Fellows of the Royal Society including George Pearson and Everard Home, both of whom taught Carpue at St. George's, as well as John Heaviside who referred his first nasal loss patient to Carpue's. The certificate was read 10 times and voted on February 17, 1817. It reads: “Joseph Constantine Carpue Esquire of Dean [Street] Soho Westminster Member of the Royal College of Surgeons teacher of anatomy late Surgeon of the Royal York Hospital Author of several works of Anatomy and Physiology being desirous of the Honor of becoming a Fellow of the Royal Society we whose names are undersigned do from our personal knowledge recommend him as a Gentleman well deserving of that honor and likely to be a valuable Member.” Courtesy of The Royal Society, London.

The best measurements of Carpue's relevance are the events that immediately followed the publication of his book. Carpue's book was published in early 1816 and its importance was rapidly recognized. By July of that year he was nominated for membership in the Royal Society, and he was elected to membership on February 13, 1817. (Figure 6)^6^ That same year, Michaelis translated Carpue's book into German.^19^ In 1818, von Graefe, who was professor of surgery at Berlin, published his own book on nasal reconstruction in both Latin, *de Rhinoplastice,* and German, *der Rhinoplastik.*^20,21^ This was the first time that the root plastik was adopted to describe our specialty, which would eventually be named plastic surgery by Zeis.^22^

In the history of science there are well-established standards for ascribing priority that have been tried to analyze plastic surgery.^23^ Sir Richard Owen and Sir Francis Darwin succinctly stated these standards:

Owen: “He becomes the true discoverer who establishes the truth; and the sign of the proof is the general acceptance.”^24^

Darwin: “In science the credit goes to the man who convinces the world, not to the man to whom the idea first occurs.”^25^

In his foreword to Michaelis' translation of Carpue's book, von Graefe clearly showed why Carpue deserved credit for introducing plastic surgery to the modern world. Von Graefe wrote:

Carpue earned for himself the merit of being the first in our part of the world to try the operation and to make known the details of its successful accomplishment … He was the first physician to perform the operation, complete it successfully, report it instructively and then open the way for scientifically minded physicians.^19(vii-x)^

## CONCLUSION

Even today many advances in plastic surgery are the result of chance rather than a product of a process that is ethical, logical, and objective. Carpue launched the modern era of plastic surgery, and he did so in an ethical, logical, and objective manner. While plastic surgery has changed in two hundred years, the principles that Carpue followed remain valid.

## 

### Disclosures

The author declared no potential conflicts of interest with respect to the research, authorship, and publication of this article.

### Funding

The open access fee was paid by the Wellcome Library Open Access Fund. No other financial support was received for the research, authorship, and publication of this article.
